# 1089. Evaluation of breakthrough infections in solid organ transplant recipients: the prospective CONTRAST cohort

**DOI:** 10.1093/ofid/ofac492.929

**Published:** 2022-12-15

**Authors:** Beatrice Tazza, Cecilia Bonazzetti, Natascia Caroccia, Dino Gibertoni, Maria Cristina Morelli, Mariarosa Tamé, Marco Busutti, Luciano Potena, Elena Salvaterra, Chiara Gamberini, Pierluigi Viale, Tiziana Lazzarotto, Maddalena Giannella

**Affiliations:** Department of Medical and Surgical Sciences, Alma Mater Studiorum University of Bologna, Rome, Emilia-Romagna, Italy; Department of Medical and Surgical Sciences, Alma Mater Studiorum University of Bologna, Rome, Emilia-Romagna, Italy; Department of Medical and Surgical Sciences, Alma Mater Studiorum University of Bologna, Rome, Emilia-Romagna, Italy; Department of Medical and Surgical Sciences, Alma Mater Studiorum University of Bologna, Rome, Emilia-Romagna, Italy; Internal Medicine Unit for the Treatment of Severe Organ Failure, IRCCS Policlinic S. Orsola, Bologna, Emilia-Romagna, Italy; IRCCS Policlinico di Sant'Orsola, Bologna, Emilia-Romagna, Italy; Alma Mater Studiorum - IRCCS Policlinico di Sant'Orsola, Bologna, Emilia-Romagna, Italy; Alma Mater Studiorum - IRCCS Policlinico di Sant'Orsola, Bologna, Emilia-Romagna, Italy; Alma Mater Studiorum - IRCCS Policlinico di Sant'Orsola, Bologna, Emilia-Romagna, Italy; Department of Medical and Surgical Sciences, Alma Mater Studiorum University of Bologna, Rome, Emilia-Romagna, Italy; Infectious Diseases Unit, Department of Medical and Surgical Sciences, Policlinico Sant'Orsola Malpighi, University of Bologna, Bologna, Italy, Bologna, Emilia-Romagna, Italy; Department of Medical and Surgical Sciences, Alma Mater Studiorum University of Bologna, Rome, Emilia-Romagna, Italy; Department of Medical and Surgical Sciences, Alma Mater Studiorum University of Bologna, Rome, Emilia-Romagna, Italy

## Abstract

**Background:**

Solid organ transplant (SOT) recipients are at higher risk than general population for complicated COVID-19 course. Moreover COVID-19 vaccination in this setting is associated with a suboptimal immune response. However, the impact of this finding on the risk of breakthrough infection (BI) in SOT recipients has to be yet determined.

**Methods:**

Single-center prospective longitudinal cohort of adult SOT recipients who received three doses of mRNA COVID-19 vaccine between February and December 2021 and were followed up to March 30 2022. Patients were tested for antibody response at several timepoints (1 st dose, 2 nd dose, 3±1 month after 1 st dose, and 1 month after 3 rd dose). Main endpoints were: i) BI defined as laboratory confirmed SARS-CoV2 infection diagnosed ≥14 day after 2 nd dose; ii) positive antibody response (AbR) defined as anti-rapid binding domain titer ≥5 U/ml determined by Elecsys Anti-SARS-CoV-2 ECLIA assay (Roche Diagnostics, CH), the last available determination before BI was considered.

**Results:**

Study cohort consists of 642 SOT (277 kidney, 191 liver, 144 heart, 37 lung) recipients: 63.9% males, median age 54 ± 14.5 years. Of them, 111 (17.8%) developed BI, BI rates were 19.9%, 18.1%, 15.2% and 10.8% among liver, heart, kidney and lung transplant recipients, respectively. Positive-AbR was observed in 60% of all patients, but rates varied from 8.7% to 91.3% among patients with BI and without BI, respectively. Predictors of BI infection at multivariable analysis were liver (vs. other grafts) transplant (OR 2.98, 95%CI 1.47-6.03), mycophenolate (1.63, 0.92-2.88) and steroids (1.8, 1.05- 3.33), while positive-AbR (0.61, 0.35-1.04) and age (0.97, 0.95-0.99) were protective. On the other hand, liver transplant (1.94, 1.02-3.69), time from transplant (1.09, 1.05-1.21), and Moderna vaccine (2.32, 1.46-3.70) were associated with positive-AbR, while age (0.97, 0.95-0.98), heart transplant (0.56, 0.33-0.96), mycophenolate (0.65, 0.39-1.06) and steroids (0.39, 0.23-0.65) with lower probability of positive-AbR.

**Conclusion:**

Although associated with positive-AbR, liver transplant and younger age were also BI predictors, suggesting the importance of social factors and the controversial role of immune monitoring.

**Disclosures:**

**All Authors**: No reported disclosures.

